# Global Patterns of Trends in Incidence and Mortality of Dengue, 1990–2019: An Analysis Based on the Global Burden of Disease Study

**DOI:** 10.3390/medicina60030425

**Published:** 2024-03-01

**Authors:** Irena Ilic, Milena Ilic

**Affiliations:** 1 Faculty of Medicine, University of Belgrade, 11000 Belgrade, Serbia; ajrini10@gmail.com; 2 Department of Epidemiology, Faculty of Medical Sciences, University of Kragujevac, 34000 Kragujevac, Serbia

**Keywords:** dengue, epidemiology, incidence, mortality, trends

## Abstract

*Background and Objectives*: Dengue is an important public health concern that warrants an examination of the longer-term global trends of its disease burden. The aim of this study was to assess the trends in dengue incidence and mortality worldwide over the last three decades. *Materials and Methods*: A descriptive epidemiological study was carried out, investigating the trends in the incidence and mortality of dengue from 1990 to 2019. The dengue incidence and mortality data were obtained from the Global Burden of Disease study database. Trends were examined using joinpoint regression analysis. *Results*: Globally, there were 56.7 million new cases of dengue reported in 2019: the disease was diagnosed in 27.4 million males and 29.3 million females. A total of 36,055 (18,993 males and 17,032 females) related deaths were reported worldwide in 2019. In both sexes, about 60% of new cases were recorded in the South-East Asia region (16.3 million in males and 17.4 million in females). Globally, the incidence of dengue exhibited an increasing tendency from 1990 to 2019 in both sexes (equally, by 1.2% per year). A significantly decreasing trend in the mortality of dengue was recorded only in females (by −0.5% per year), while an increasing trend was observed in males (by +0.6% per year). *Conclusions*: The rise in the number of new dengue cases and deaths in the world in the last several decades suggests a need for implementing more effective prevention and management measures.

## 1. Introduction

The World Health Organization declared dengue one of the “top ten threats to global health in 2019” [1]. Dengue is one of the major vector-borne diseases and presents an important public health issue in the world [2]. In recent decades, a steep and rapid rise in the number of dengue cases across the world has been raising concerns [3,4]. Dengue exists throughout the world and occurs endemically, sporadically, or in the form of epidemics. According to the World Health Organization (WHO), dengue is endemic in more than 100 countries, mainly in tropical and subtropical areas (South-East Asia, Africa, the Western Pacific, the Eastern Mediterranean, and the Americas), with Asia accounting for about 70% of the global disease burden [2]. Additionally, dengue is frequently transported across remote areas by infected travelers [5,6,7], and the presence of vectors in these new areas represents a potential risk for local transmission to be established [8].

Dengue is an acute, transmissible (mosquito-borne) infectious disease caused by the dengue virus [2]. The dengue virus belongs to the genus *Flavivirus*, of the family *Flaviridae*, with four different serotypes [9]. The dengue virus has a spherical structure, with a diameter of approximately 50 nm, and its genome is a single strand of RNA [10]. There are four closely related dengue viruses (serotypes) named DEN-1, DEN-2, DEN-3, and DEN-4. During the last few decades, all four viruses have spread widely, and all circulate together in the same regions around the world [11]. The spread of the dengue virus includes sylvatic cycles (between *Aedes* mosquitoes and nonhuman primates), transmission between humans and *Aedes* mosquitoes, as well as vertical transmission from mosquito to mosquito [12]. Humans with dengue are not contagious.

The sources and hosts of the virus are humans and African and Asian primates. The virus is transmitted to humans through the bites of infected female mosquitoes (*Aedes aegypti* and *Aedes albopictus*). Incubation lasts 5–8 days. Although most infections are asymptomatic, clinical features have two forms: classic dengue fever and hemorrhagic dengue fever [13]. The hemorrhagic form is accompanied by a drop in blood pressure and cardiocirculatory collapse, purpura, petechiae on the skin and mucous membranes, bleeding from the digestive tract, and hemorrhagic pneumonia. In the case of an unfavorable course of disease, dengue shock syndrome can occur with a fatal outcome. Today, there is no effective specific antiviral therapy. Dengue can be lethal, and the case fatality rate reaches up to 20% of those with severe dengue [1,2,13].

After recovering from infection with one dengue serotype, a person acquires long-term serotype-specific immunity [9,14]. In addition, a person is protected from infection caused by the other three serotypes, but only for two to three months after the first dengue infection. After that, a person can become infected with any of the other three serotypes. A person can be infected with the dengue virus several times during their life, either consecutively or simultaneously with two different serotypes. However, for a person who has gotten over dengue fever, a large issue is the occurrence of any subsequent infection with the dengue virus, because it can put a person at high risk for the occurrence of the most severe forms of dengue infection compared to persons not previously infected with the virus [15]. A special problem in the epidemiology of dengue fever is frequent infection with the dengue virus in pregnant women, whose newborns have antibodies from the mother and are susceptible to dengue hemorrhagic fever if they are infected with one of the other three serotypes [16]. Also, in endemic regions, 90% of dengue hemorrhagic fever cases occur in children who have multiple dengue virus infections [17].

Any of the four dengue viruses can cause a wide range of symptoms, including those that are extremely mild to those that are extremely severe and can lead to death. However, dengue and dengue hemorrhagic fever are pathogenetically, clinically, and epidemiologically distinct [18]. One of the possible explanations for the occurrence of more severe forms of dengue, such as dengue hemorrhagic fever and dengue hemorrhagic shock syndrome, includes immunopathological mechanisms that lead to exacerbation by pre-existing immunity in persons following subsequent infection with a related dengue virus (i.e., antibody-dependent enhancement) [19]. In contrast, this process is not present in dengue fever. Therefore, antibodies to previous infections with one dengue serotype may interfere with the immune response to the current serotype, increasing the risk of developing a severe form of dengue infection.

Prevention and control of dengue fever are based on vaccines and on vector control. In order for a vaccine against dengue fever to be successful and effective, it must enable the creation of adequate immunity against all four dengue serotypes in order to avoid the risk of immunopathological phenomena, i.e., antibody-dependent enhancement due to subsequent exposure to wild strains [20]. Decisions about implementing the live attenuated dengue vaccine CYD-TDV requires careful assessment at the country level, based on the WHO position paper [2,21]. Integrated surveillance and vector management are of crucial importance in the prevention of dengue at present. A dengue case is subject to mandatory reporting in case of suspicion, illness, or death [2,7].

According to a report by the WHO, about 40% of the world’s population (3.6 billion people) is at risk of dengue fever, and there are around 390 million infections a year [2]. A high number of cases occurs in Asia, mainly during the rainy seasons in countries such as Indonesia, Bangladesh, and India [2,4]. People of all ages who are exposed to infected mosquitoes are at risk of developing dengue, whether they live in an area with a risk or travel to an area with a risk of dengue. In endemic areas (primarily South-East Asia), dengue occurs mainly in children, with dengue hemorrhagic fever being the major cause of death among children, with a case fatality rate of 3 to 10 percent [22]. Dengue outbreaks show seasonal patterns, with peak cases occurring during and after the warm and rainy seasons. Circumstances that contribute to the epidemic spread of the disease include an increase in mosquito populations caused by favorable temperature and air humidity that correspond to the development of the mosquito life cycle, but also numerous social factors, such as population density, unplanned urbanization, migration, insufficient implementation of preventive vector control measures in the community, and lack of professional staff, as well as the knowledge, attitudes, and practices of the population regarding dengue fever [23].

Dengue poses a huge economic and disease burden in South-East Asia: a systematic literature review showed that, over the decade of 2001–2010, the annual economic burden due to this disease was USD 950 million, while the annual number of disability-adjusted life years (DALYs) was 372 DALYs per million inhabitants [24]. Recent outbreaks of dengue in Pakistan, Brazil, Indonesia, and Peru are among the largest that these countries have faced [2,7]. Although a high number of cases occurs in the rainy seasons, now the season of dengue is lengthening significantly in endemic countries, and the disease is spreading to more temperate countries (Nepal and Afghanistan) [7,25,26]. The changes in the epidemiology of dengue should be taken into account to properly prevent its further spread. For this, it is essential to examine temporal trends and the frequency of the disease. The aim of this study was to assess the trends in dengue incidence and mortality in the world over the last three decades.

## 2. Materials and Methods

### 2.1. Study Design

A descriptive epidemiological study of global trends in incidence and mortality due to dengue from 1990 to 2019 was carried out.

### 2.2. Data Source

Data on incidence and mortality due to dengue were extracted from the Global Burden of Disease (GBD) 2019 study [27]. The GBD 2019 study represents systematically and comprehensively estimated causes of diseases and deaths and provides rigorous and comparable measurements of important health problems around the world, based on a combination of relevant publicly available and restricted data sources, including vital statistics, verbal autopsies, surveillance data, household surveys, censuses, and civil registrations [27]. The GBD 2019 estimates for dengue disease represent cases of clinically apparent illness (dengue fever, dengue hemorrhagic fever, dengue shock syndrome, and resulting chronic fatigue syndrome) and death. For dengue, the GBD cause list covered codes A90–91 (based on the 10th revision of the International Classification of Diseases) and code 061 (based on the 9th revision of the International Classification of Diseases).

Age-standardized rates (ASRs) of incidence and mortality were used to assess the trends between 1990 and 2019. The ASRs (expressed per 100,000 persons) were calculated using the direct method, using the world standard population developed for the GBD study [27].

Besides the global trends, incidence and mortality due to dengue were presented within the six WHO regions (Africa, the Americas, South-East Asia, Europe, the Eastern Mediterranean, and the Western Pacific). Also, incidence and mortality due to dengue were presented for 204 countries and territories worldwide in 2019 [27]. In addition, global trends in age- and sex-specific rates in incidence and mortality were presented. Subgroup analyses were also performed: the age groups were divided into four strata (0–9/10–24/25–49/50–69/70+ years) for males and females separately.

### 2.3. Statistical Analysis

The magnitude and direction of temporal trends of incidence and mortality were assessed using joinpoint regression analysis (Joinpoint regression software, Version 4.8.0.1—April 2020, available through the Surveillance Research Program of the US National Cancer Institute), proposed by Kim et al. [28]. Joinpoint regression analysis fits models to data that allow testing and identification of whether a change in a trend is statistically significant. The permutation test was applied, and each permutation test was estimated using the Monte Carlo method. The joinpoint regression analysis estimated the average annual percent change (AAPC) in rates, with respective 95% confidence intervals (95% CIs), as a summary measure of the trend [29]. For describing the direction of trends, the terms “significant increase” or “significant decrease” were used in order to signify whether the slope of a trend was statistically significant (on the basis of the statistical significance of the AAPC compared to zero). Disparities in trends in incidence and mortality due to dengue according to sex and age were tested using the comparability test [30]. The comparability test determines whether two line regression functions are parallel (test of parallelism). A *p* value < 0.05 was considered statistically significant. 

## 3. Results

### 3.1. Incidence and Mortality Due to Dengue: 2019 Results

**Figure 1:**
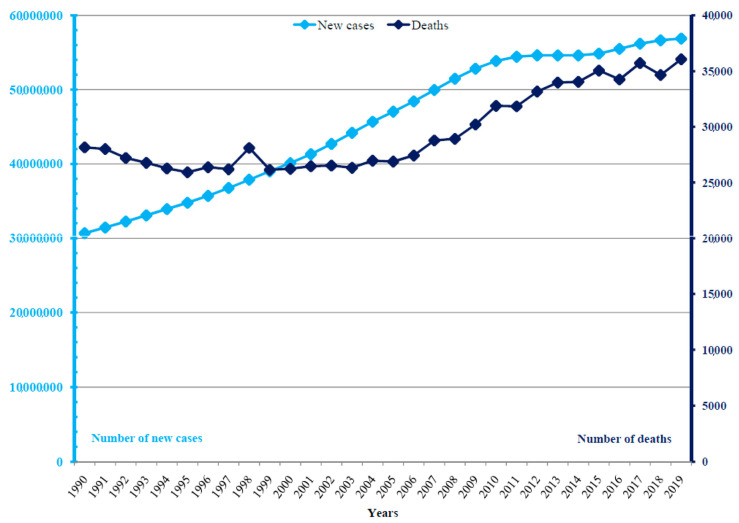
Dengue: numbers of new cases and deaths in the world, 1990–2019.

**Figure 2:**
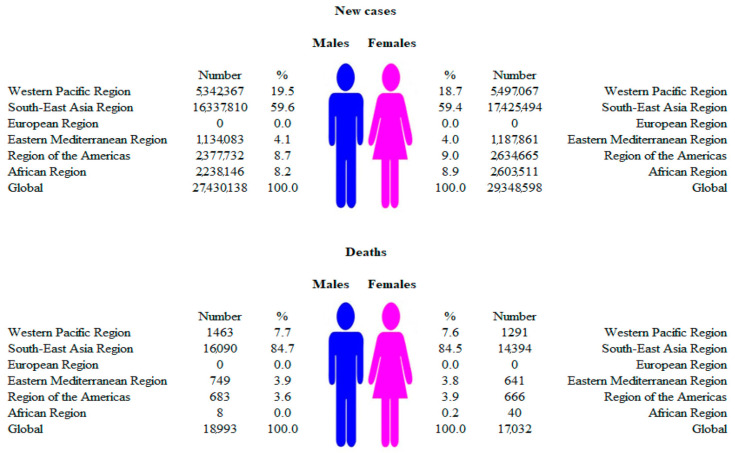
Numbers of new cases and deaths due to dengue, by WHO regions and sexes, 2019.

Globally, there were 56.7 million new cases of dengue reported in 2019: it was diagnosed in 27.4 million (48%) males and 29.3 million (52%) females (Figure 1 and Figure 2). A total of 36,055 (18,993 males and 17,032 females) related deaths were reported worldwide in 2019. The frequency of dengue increased greatly between 1990 and 2019, with the number of new cases almost doubling (from 30.7 million cases in 1990 to 56.9 million cases in 2019), and the number of deaths increased from 28,151 in 1990 to 36,055 in 2019.

In males, most of the new cases of dengue (16.3 million; about 60% of the total) were recorded in the South-East Asia region, followed by the Western Pacific region (5.3 million; about 20%), in 2019 (Figure 2). Similarly to males, a dominant proportion of new cases of dengue in females was evident in the South-East Asia region (17.4 million; about 60% of the total), followed by the Western Pacific region (5.5 million; about 20%). In the European region, not a single autochthonous case of new illness or death from dengue was registered in either men or women. In 2019, most of the deaths from dengue were recorded in the South-East Asia region, both in males (16,090; about 85% of the total deaths in males) and females (14,394; about 85% of the total deaths in females).

**Figure 3:**
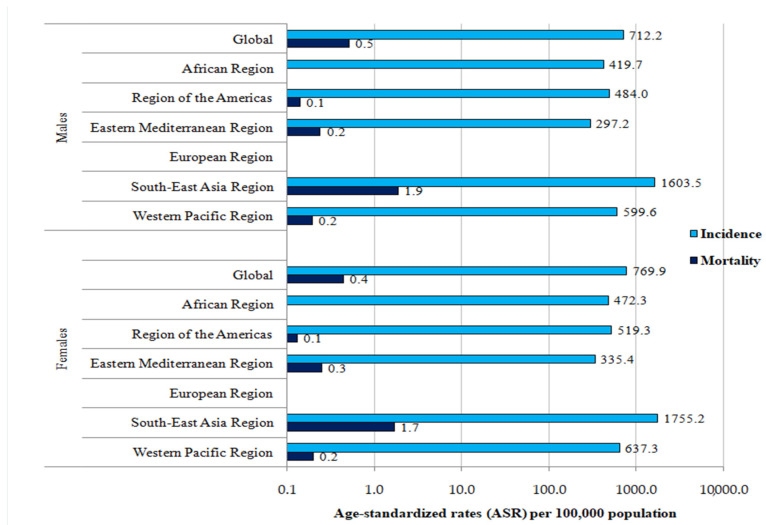
Incidence and mortality (ASRs) of dengue (globally and by WHO regions), by sex, 2019.

The global ASR of incidence of dengue was 712.21 per 100,000 persons in males and 769.95 per 100,000 persons in females (Figure 3). Both in males and females, the highest ASRs of incidence were found in the South-East Asia region (1603.47 per 100,000 in males and 1755.17 per 100,000 in females), which were about three to five times higher than in all other regions, with the exception of the European region, where no new cases of dengue were registered.

The global ASR of mortality from dengue was 0.52 per 100,000 persons in males and 0.44 per 100,000 persons in females (Figure 3). Both in males and females, the highest ASRs of mortality were found in the South-East Asia region (1.88 per 100,000 in males and 1.68 per 100,000 in females), which were about three to five times higher than in all other regions, with the exception of the European region, where no deaths due to dengue were registered.

**Figure 4:**
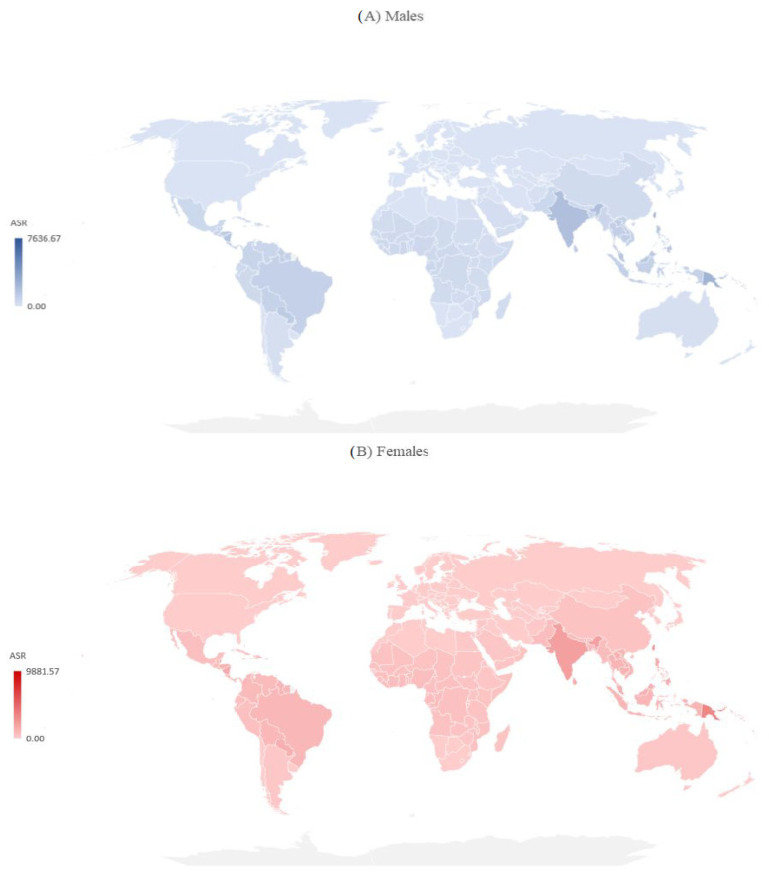
Incidence of dengue (globally and by WHO regions), by sex, 2019.

**Figure 5:**
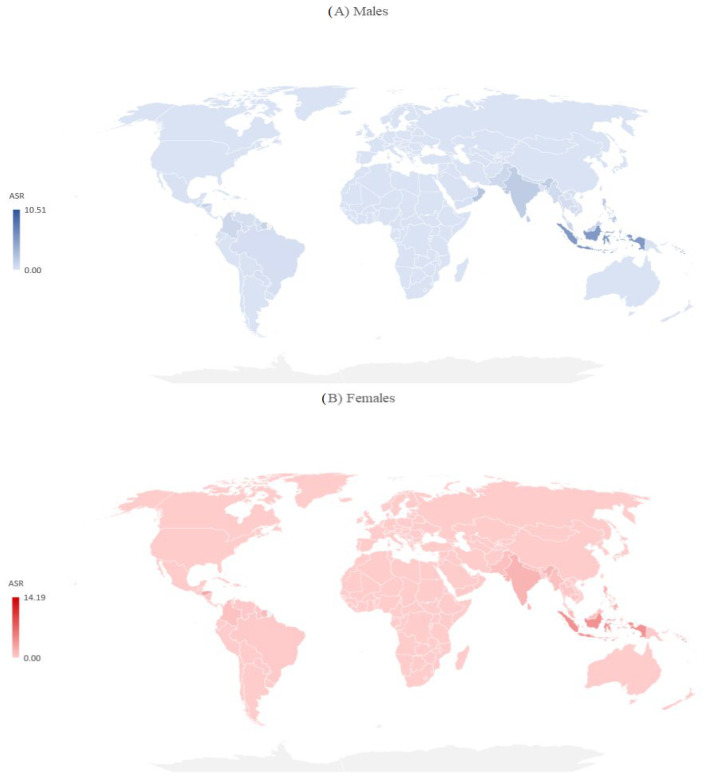
Mortality due to dengue (globally and by WHO regions), by sex, 2019.

There are significant international differences in the incidence and mortality of dengue by sex in 2019 (Figure 4 and Figure 5). Both in males and females in 2019, the incidence ASRs were the highest in Kiribati, the Northern Mariana Islands, the Seychelles, and Niue (equally, about 7.500 per 100,000 in males and about 9.000 per 100,000 in females), while the lowest ASRs were reported in the United States of America (7.11 per 100,000 in males, 7.19 per 100,000 in females) (Figure 4).

Both in males and females, the ASRs of mortality varied greatly across countries, and the mortality rate was highest in the Solomon Islands (10.51 per 100,000 in males, 14.19 per 100,000 in females), followed by Indonesia (5.52 per 100,000 in males, 4.02 per 100,000 in females) (Figure 5).

### 3.2. Trends of Dengue Incidence and Mortality, 1990–2019

**Figure 6:**
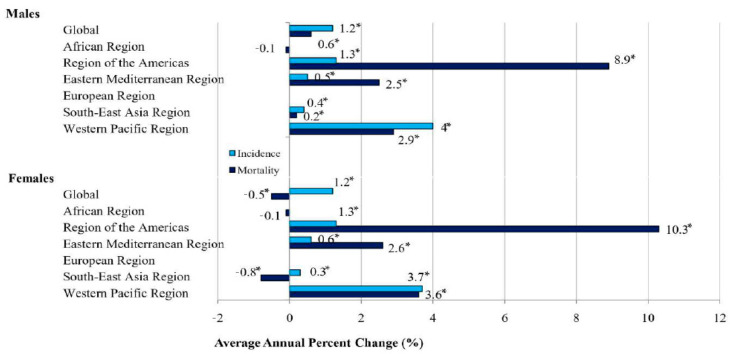
Trends in ASRs for incidence and mortality of dengue, by sex, 1990–2019: a joinpoint analysis. * Statistically significant trend (*p* < 0.05).

Globally, from 1990 to 2019, the ASRs for the incidence of dengue showed a significantly increasing tendency both in males and females (equally by 1.2% per year) (Figure 6, Table 1). According to the comparability test, the trends in incidence due to dengue in males and in females were not parallel (the final selected model rejected parallelism, *p* < 0.05). A significantly decreasing trend in the mortality of dengue was recorded only in females (AAPC = −0.5%, 95% CI = −0.7 to −0.3), while an increasing trend was observed in males (AAPC = +0.6%, 95% CI = 0.5 to 0.8). According to the comparability test, trends in mortality due to dengue in males and in females were not parallel (the final selected model rejected parallelism, *p* < 0.05).

When the incidence trend of dengue was analyzed in six WHO regions, significantly increasing trends were observed both in males and in females in four regions: in the Western Pacific (by 4.0% and by 3.7% per year, respectively), the Americas (both equally, by 1.3% per year), the Eastern Mediterranean (by 0.5% and 0.6% per year, respectively), and South-East Asia (by 0.4% and by 0.3% per year, respectively).

The mortality trends for dengue were significantly increasing both in males and in females in three regions: the Americas (by 8.9% and by 10.3% per year, respectively), the Western Pacific (by 2.9% and by 3.6% per year, respectively), and the Eastern Mediterranean (by 2.5% and by 2.6% per year, respectively). However, while in the South-East Asia region a significantly increased trend of dengue mortality in males (by 0.2% per year) was observed, a significantly decreasing trend was observed in females (by −0.8% per year).

The only exception was the region of Africa, with a stable trend of dengue mortality both in males and females (both equally, by −0.1% per year).

Globally, the age-specific incidence rates of dengue in females were higher than in males in all age groups in 2019 (Table 1). A significant increase in incidence rates due to dengue was noted in all age groups in both males and females. In almost all age groups (≥10 years), a significantly increasing trend in the mortality of dengue was recorded both in males and females. A significantly decreasing trend in the mortality of dengue was recorded only in the youngest age group (0–9 years) in both males (by −1.4% per year) and females (by −3.1% per year).

## 4. Discussion

This study showed that the global ASRs of incidence and mortality due to dengue have increased significantly from 1990 to 2019. A significantly decreasing trend in the mortality of dengue was recorded only in females, while a rising trend was observed in males.

The global incidence and mortality from dengue increased significantly between 1990 and 2019, and this trend was present in almost all regions and analyzed age groups. Some previous studies have presented similar patterns of dengue incidence and mortality [26,31,32,33,34]. The rapid rise in the global burden of dengue in the last decades could be related to population growth, aging, urbanization, warming climates, and increased human mobility in much of the world [2,7,34]. Additionally, large international differences (from region to region and country to country, as well as in the magnitude and direction of trends) in incidence and mortality rates due to dengue could be partly due to socio-economic development. A recent study showed that most of the countries with the highest burden of dengue fever were in areas with low and medium socio-demographic indexes (composite averages of the rankings of incomes per capita, average educational attainments, and fertility rates), while the burden was relatively low in developed countries [3]. Inequalities in the development and availability of public health infrastructure, as well as vector control, could be linked to differences in the geographic distribution and frequency of dengue throughout the world [2,31]. Furthermore, the concern about under-representation and under-reporting of dengue due to discrepancies in surveillance systems exists all over the world, especially in countries with limited resources [35]. Also, many cases of dengue fever may be misdiagnosed and reported as other febrile illnesses. In addition, it is known that a vast majority of dengue cases are asymptomatic or mild and self-managed, so the question is how much such cases contribute to the spread of the infection in the environment and to remote areas [36].

In summary, the incidence and mortality of dengue correspond to the geographical distribution patterns of the principal vector, *Aedes aegypti*. The dengue virus mainly and most often occurs in tropical countries in South-East Asia, South Asia, and South America, where there are high numbers of infected mosquitoes. Although the South-East Asia region remains a region of concern regarding dengue, the virus has a high potential to spread to new areas. Some studies indicated that in addition to the influence of mean temperature values, the effect of the diurnal temperature range on the behavior of *Aedes aegypti* should be taken into account, while climate change projections suggest that large increases are expected by the end of this century in temperate Northern Hemisphere regions [37,38]. In 2022, and as of 23 August, in the Americas region there were 2,141,240 reported dengue cases, while the country reporting the most cases was Brazil (1,910,657) [7]. Environmental changes (El Niño, La Niña, precipitation, and deforestation) were associated with dengue infection in humans in Brazil since 2010 [39]. Finally, a lack of effective specific antiviral therapy and issues regarding the implementation of vaccines against dengue are of key importance for the unfavorable global and regional trends in incidence and mortality due to dengue [1,21]. Also, many reports indicated that, in recent years, dengue has been spreading to new regions, such as Europe, where local transmission and several epidemics have been registered [40]. Sporadic indigenous cases of dengue occasionally occur in Europe (Spain and France), following the introduction of the dengue virus by viremic travelers to areas where *Aedes albopictus* has become established [40]. In 2010, two autochthonous cases of dengue fever were reported in southern France, diagnosed for the first time ever in Europe [6]. At the same time, local transmission of dengue fever was reported for the first time on several islands in the south of Croatia [8]. In addition, several hundred imported cases of dengue fever have been detected in some European countries in the last two decades [40].

Our study confirmed the findings of some authors that the age-standardized incidence rate is slightly higher in females than in males, but the results were not consistent [26,41]. Gender differences could be related to sex-related biological differences and differences in exposure, occupation, and socio-economic status, while there are no findings about the mode of dengue virus transmission depending on gender [41]. Contrary to the ASRs for incidence, the ASRs for mortality due to dengue were slightly higher in males than in females. The mentioned differences in mortality by gender could be at least partially related to some biological, innate differences or related to sex differences in genetics and hormones.

The increasing trend in the dengue burden worldwide indicates that this global threat to public health warrants the development of more effective prevention measures (including the development of vaccines as well as vector control) and the development of specific treatment interventions. Two types of vaccine against dengue infection have been approved and are available, and both are tetravalent live vaccines [42,43]. In Mexico, in 2015, Dengvaxia, the first dengue vaccine to be licensed, was approved for use only in individuals of 6 through 16 years of age with laboratory-confirmed previous dengue infection and living in endemic areas [42]. In 2022, the European Medicines Agency approved the Qdenga vaccine for adults, adolescents, and children from four years of age for use in the European Union and in the United Kingdom, Brazil, Argentina, Indonesia, and Thailand [43]. General measures to prevent dengue infection include vector control activities in the community (destruction of larval habitats and spraying of insecticides to kill adult mosquitoes) and reducing the risk by using personal protective equipment (suitable clothing, mosquito nets, insect repellent, etc.).

In addition, reducing inequality in access to healthcare worldwide and reducing socio-economic inequalities is necessary in order to reduce the dengue burden [15]. For health policy makers, understanding the burden of dengue fever is essential for the rational allocation of limited resources.

### Strengths and Limitations

This study has presented comprehensive global trends in the incidence and mortality of dengue in the last three decades. Notably, this study represents a substantial improvement compared to some previous studies, in which fewer countries were considered, shorter time periods were observed, and only linear trend assessments were performed. However, several sources of limitations in this study should be taken into account. First, the accuracy of all estimates generally depends on the availability and quality of data, that is, the question of data reliability can always be raised. Although most dengue cases in Europe were reported as travel-related cases, in some countries (such as France, Portugal, etc.), locally acquired cases were reported [44,45]. Further efforts in providing reliable data, that is, complete and accurate data, which are the key basis for disease prevention and control worldwide, are necessary. Also, a limitation specific to evaluating dengue-related deaths can lead to under-estimation of the incidence and mortality of dengue depending on the development and availability of health services. Despite these shortcomings, this study provides useful insights into the variations in the mortality of dengue worldwide and could provide help for health authorities and policy makers to develop more effective dengue prevention strategies based on reliable estimates of the incidence and mortality of dengue.

## 5. Conclusions

This study highlights the current situation regarding dengue trends and distribution worldwide and can be used to improve surveillance and reduce the burden of the disease. The rise in the number of new dengue cases and deaths across the world in the last several decades suggests a need for implementing more effective prevention and management measures.

## Figures and Tables

**Table 1:** Joinpoint regression analysis: global trends * in incidence and mortality rates (per 100,000) due to dengue, by age and sex, 1990–2019.

Age	Males			Females		
	Age-Specific Rates		AAPC (95% CI)	Age-Specific Rates		AAPC (95% CI)
	1990	2019		1990	2019	
	Incidence					
0–9	573.18	638.94	+0.5 * (0.2 to 0.7)	642.01	699.92	+0.4 * (0.1 to 0.7)
10–24	673.84	944.35	+1.4 * (1.2 to 1.5)	744.05	1004.60	+1.2 * (1.1 to 1.4)
25–49	476.92	680.25	+1.5 * (1.4 to 1.7)	538.00	738.80	+1.4 * (1.2 to 1.6)
50–69	409.88	530.57	+1.0 * (0.9 to 1.1)	435.99	599.59	+1.2 * (1.1 to 1.3)
70+	425.57	607.24	+1.4 * (1.3 to 1.5)	381.84	631.87	+2.1 * (1.9 to 2.2)
	Age-standardized rates					
All ages	532.12	712.21	+1.2 * (1.0 to 1.4)	584.29	769.95	+1.2 * (1.0 to 1.4)
	Mortality					
0–9	1.19	0.70	−1.4 * (−1.6 to −1.2)	1.83	0.71	−3.1 * (−3.2 to −2.9)
10–24	0.21	0.29	+1.2 * (1.0 to 1.4)	0.20	0.21	+0.2 * (0.1 to 0.3)
25–49	0.20	0.32	+2.1 * (1.9 to 2.2)	0.17	0.24	+1.5 * (1.2 to 1.7)
50–69	0.34	0.54	+2.0 * (1.8 to 2.3)	0.27	0.46	+2.3 * (1.9 to 2.6)
70+	0.94	1.65	+2.3 * (2.0 to 2.6)	0.66	1.61	+3.5 * (3.2 to 3.9)
	Age-standardized rates					
All ages	0.47	0.52	+0.6 * (0.5 to 0.8)	0.54	0.44	−0.5 * (−0.7 to −0.3)

* Statistically significant trend (*p* < 0.05). AAPC, for full period presented AAPC (average annual percent change); CI = confidence interval. Source: GBD estimates [27].

## Data Availability

Data are contained within the article.
